# Multimodal Strategies for the Diagnosis and Management of Refractory Congestion. An Integrated Cardiorenal Approach

**DOI:** 10.3389/fphys.2022.913580

**Published:** 2022-07-08

**Authors:** Diana Rodríguez-Espinosa, Joan Guzman-Bofarull, Juan Carlos De La Fuente-Mancera, Francisco Maduell, José Jesús Broseta, Marta Farrero

**Affiliations:** ^1^ Department of Nephrology and Renal Transplantation, Hospital Clínic of Barcelona, Barcelona, Spain; ^2^ Department of Cardiology, Hospital Clínic of Barcelona, Barcelona, Spain

**Keywords:** heart failure, refractory congestion, diuretic resistance, peritoneal dialysis, extracorporeal ultrafiltration

## Abstract

Refractory congestion is common in acute and chronic heart failure, and it significantly impacts functional class, renal function, hospital admissions, and survival. In this paper, the pathophysiological mechanisms involved in cardiorenal syndrome and the interplay between heart failure and chronic kidney disease are reviewed. Although the physical exam remains key in identifying congestion, new tools such as biomarkers or lung, vascular, and renal ultrasound are currently being used to detect subclinical forms and can potentially impact its management. Thus, an integrated multimodal diagnostic algorithm is proposed. There are several strategies for treating congestion, although data on their efficacy are scarce and have not been validated. Herein, we review the optimal use and monitorization of different diuretic types, administration route, dose titration using urinary volume and natriuresis, and a sequential diuretic scheme to achieve a multitargeted nephron blockade, common adverse events, and how to manage them. In addition, we discuss alternative strategies such as subcutaneous furosemide, hypertonic saline, and albumin infusions and the available evidence of their role in congestion management. We also discuss the use of extracorporeal therapies, such as ultrafiltration, peritoneal dialysis, or conventional hemodialysis, in patients with normal or impaired renal function. This review results from a multidisciplinary view involving both nephrologists and cardiologists.

## 1. Introduction

The clinical course of patients with heart failure (HF) is characterized by frequent exacerbations that require urgent medical attention. It has been demonstrated that congestion, not low cardiac output, is the main reason for these acute episodes, which are associated with increased morbidity and mortality and impose a considerable economic burden on health care systems (Chioncel et al., 2017; Mullens et al., 2019).

Early detection of congestion is paramount since it allows intensification of treatment before signs and symptoms worsen, which, in turn, may prevent the need for urgent medical care (Abraham et al., 2011). Furthermore, poorly controlled congestion has also been associated with atrial and ventricular remodeling, recurrent hospital admissions, and increased mortality (Melenovsky et al., 2015; Pellicori et al., 2019). This is particularly relevant considering that up to 50% of patients admitted with acute HF (AHF) are discharged with residual congestion, which, if present, is associated with rehospitalizations and death within 6 months from discharge (Ambrosy et al., 2013).

Traditionally, clinicians have relied on the physical exam to detect congestion; however, clinical signs and symptoms are late manifestations and are neither sensitive nor specific to HF (Girerd et al., 2018). Recently, biomarkers–such as natriuretic peptides–, and imaging modalities–particularly ultrasound–have emerged as valuable aids in the early detection of congestion (Boorsma et al., 2020; Pellicori et al., 2021).

Given the central role of congestion in HF exacerbations, an adequate understanding and knowledge of diverse decongestive strategies is a must for anyone involved in the care of HF patients. Chronic kidney disease (CKD) is present in up to 51–65% of HF patients (Ahmed and Campbell, 2008) and is associated with worse outcomes, more complex management, and demands for increased monitoring (House et al., 2019). In that sense, a 2019 American Heart Association statement and the 2021 European Society of Cardiology Guidelines for the diagnosis and treatment of acute and chronic heart failure highlight the role of integrated cardiorenal management in improving quality of life and outcomes in HF (Rangaswami et al., 2019; McDonagh et al., 2021). A multidisciplinary approach is mandatory since both entities are closely related in pathophysiological and clinical terms.

This review covers the main mechanisms that lead to congestion in HF patients, the different diagnostic modalities to detect it, and the available treatment strategies from an integrated cardionephrology approach.

## 2. Heart and Kidney. A Dependent Relationship

The association between the kidney and the heart has been reported since the early 19th century when Robert Bright depicted significant cardiac structural changes in patients with advanced kidney disease (Bright, 1836). Since then, a shared etiological pathway and interdependent relationship have been described to a point where they may be referred to as the *cardiorenal vascular system* and not as each organ system separately.

As the heart pumps, blood is delivered throughout the human body. Kidneys are essential in this interaction because, depending on the pressure at which blood is provided, they will produce several hormones aiming to adjust urine output and regulate blood pressure. These hormones will act not only on blood vessels but also on cardiac muscle tissue. For instance, a low stroke volume or heart rate can lead to low cardiac output, reducing the renal blood flow and, thus, the amount of filtered plasma. Kidneys sense the reduced blood flow received and, in turn, activate the renin-angiotensin-aldosterone system (RAAS), increasing glomerular hydrostatic pressure, sodium tubular avidity, and water retention while inducing cardiac remodeling and worsening systemic hypertension. These mechanisms are required for survival in the short term; however, they lead to structural heart damage, chronic kidney disease, and volume overload when perpetuated. The latter is of current interest as it has been settled as the primary pathophysiological mechanism of that new entity described as *congestive nephropathy*. This phenomenon develops when severe volume overload significantly increases the venous system pressure, which is transmitted to the efferent arteriole, reducing the differential glomerular pressures required to generate a sufficient net glomerular ultrafiltration pressure, resulting in oliguria, increased cardiac afterload, and congestion (Jentzer et al., 2020).

Moreover, Boorsma et al. (2022) proposed another novel term, *renal tamponade,* to describe severe cases of congestive nephropathy where the rigid renal capsule, kidney surrounding fat tissue, and the peritoneal cavity exert cumulative pressure on the retroperitoneal space, limiting the space available for renal expansion. Animal models of HF showed that renal decapsulation effectively reduced kidney pressure-related injury. This might be a new vision with potential forthcoming therapeutic implications in HF by alleviating intrarenal congestion.

## 3. Classification

The National Heart, Lung, and Blood Institute Working Group has defined the cardiorenal vascular interplay as Cardiorenal syndrome (CRS) and proposed a classification based on the volume retention by the kidneys (Cardio-Renal Connections in Heart Failure and Cardiovascular Disease, 2004); however, since this definition places the heart at its center and the kidneys as the culprit, other organizations have proposed additional definitions with a broader clinical spectrum. For instance, the Acute Dialysis Quality Initiative in 2008 split the CRS into five types to easily characterize its clinical presentation for diagnostic and therapeutic purposes (Ronco et al., 2010). Type 1 or acute cardiorenal syndrome refers to AHF leading to acute kidney injury (AKI); type 2 or chronic cardiorenal syndrome, to chronic HF leading to progressive and permanent CKD; type 3 or acute renocardiac syndrome, to AKI causing AHF; type 4 or chronic renocardiac syndrome, to CKD leading to chronic HF and CKD progression; and, finally, type 5 is known as secondary CRS, and it is described as a systemic insult resulting in heart and kidney failure (e.g., sepsis, cirrhosis, or amyloidosis) (Ronco et al., 2010). Another classification proposed by Hatamizadeh et al. (2013) states that, besides cardiac pump failure, multiple body systems can lead to volume retention by the kidney and contribute to CRS. This group proposed seven categories: hemodynamic (heart failure), uremia and kidney failure, atherosclerosis, endothelial dysfunction, neurohumoral, anemia and iron disorders, mineral disorders (FGF23, phosphorus, or vitamin D disorders), and inflammatory pathways leading to malnutrition.

**FIGURE 1:**
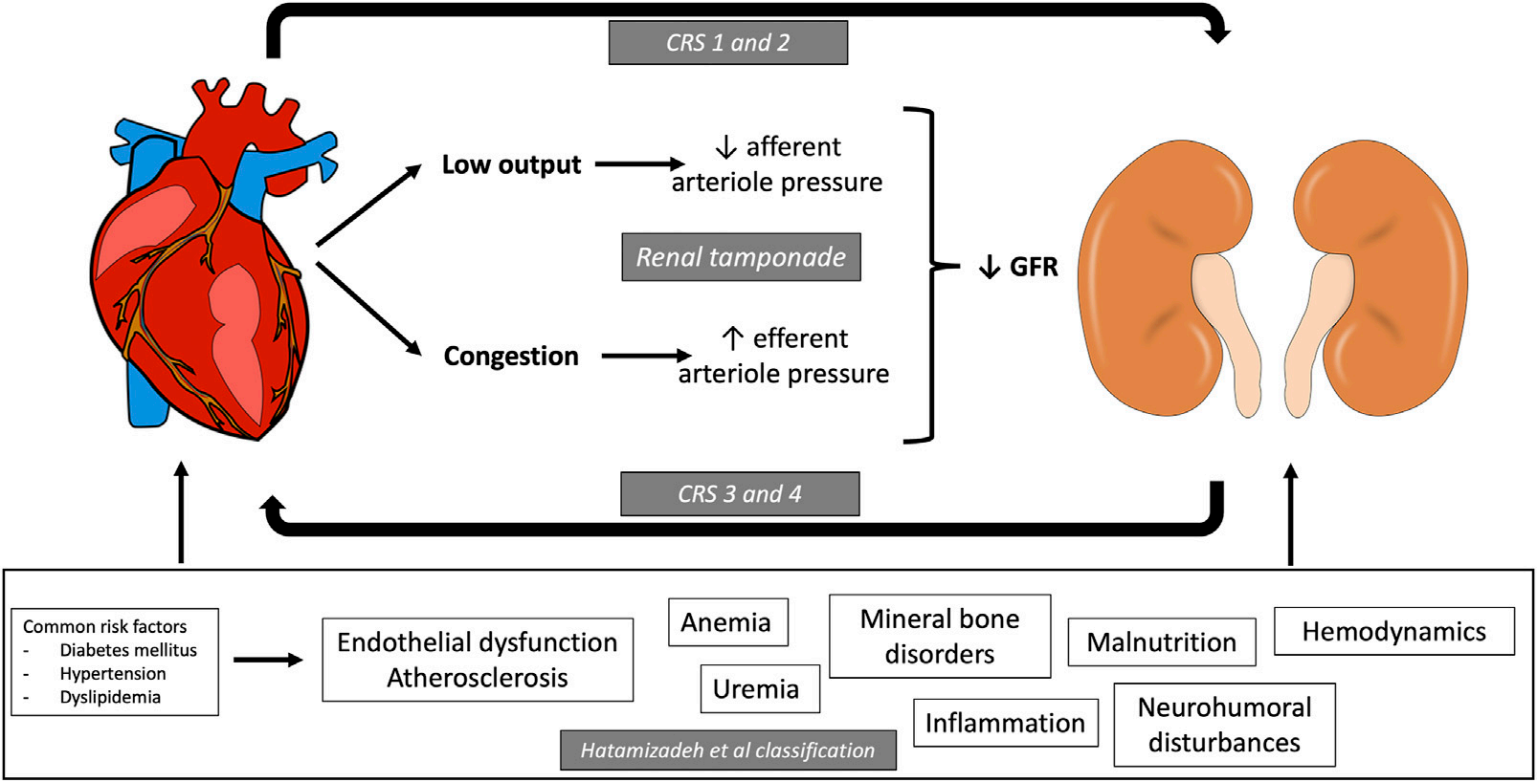
Cardiorenal pathophysiology. Interrelation between heart and kidney in the development of the different types of cardiorenal syndrome (CRS). Type 1 and type 2 CRS are described as acute (Chioncel et al., 2017) or chronic (Mullens et al., 2019) heart failure resulting in acute kidney injury (AKI) or chronic kidney disease (CKD). Type 3 and 4 CRS are known as acute and chronic renocardiac syndrome and are described as AKI (Abraham et al., 2011) or CKD (Melenovsky et al., 2015), resulting in heart failure. Type 5 is known as secondary CRS, and it is described as a systemic process resulting in heart and kidney failure. The classification by Hatamizadeh et al. proposes seven disease categories that contribute to CRS: hemodynamic (heart failure), uremia and kidney failure, atherosclerosis, endothelial dysfunction, neurohumoral, anemia and iron disorders, mineral disorders (FGF23, phosphorus or vitamin D disorders), and inflammatory pathways leading to malnutrition.

The classification of CRS is rather complex, mainly because, in many cases, it is almost impossible to identify where the process started (Figure 1). Moreover, given the importance of blood pressure and vessels in this relationship, the kidneys and the heart share vascular risk factors for disease development and progression. For instance, diabetes mellitus, hypertension, dyslipidemia, atherosclerosis, endothelial inflammation, mineral bone disorders, and anemia have been associated with both cardiovascular and renal diseases (Zannad and Rossignol, 2018). Therefore, regardless of the chosen classification, it is fundamental for nephrologists and cardiologists to understand the underlying maladaptive mechanisms that lead to the decompensation of both organ systems so a proper pathophysiological and holistic management can be offered to this complex group of patients.

## 4. Congestion

Congestion in HF is defined as the combination of signs and symptoms of extracellular fluid accumulation that result in increased cardiac filling pressure (Martens et al., 2015). Congestion and volume overload are usually misused interchangeably; however, these terms are not precisely equal. For instance, up to half of the patients with AHF barely gained weight during the weeks preceding their hospital admissions. This is because sympathetic tone decompensation in HF leads to vasoconstriction of splanchnic circulation, resulting in blood redistribution and not volume gain. This redistribution increases hydrostatic pressure and the effective circulating volume, which produce signs and symptoms of congestion (Chaudhry et al., 2007; Verbrugge et al., 2013). Volume overload, on the contrary, is due to increased neurohumoral activation that increases renal sodium and water avidity, which results in global water gain (Nijst et al., 2015). Both mechanisms increase venous return, cardiac preload, and cardiac filling pressures. Moreover, advanced HF is related to cachexia, where low plasma proteins reduce oncotic pressure and decrease plasma refilling from the interstitium. Thus, the gain in body weight could be an inaccurate measure in some cases of HF decompensation. The difference between absolute volume overload and volume redistribution may have important implications for the therapeutic approach.

Refractory congestion is defined as the persistence of symptoms that limit daily life [at least, functional class III or IV of the New York Heart Association (NYHA)] despite optimal treatment, including chronic diuretics (Nohria et al., 2002); and described as the failure to decongest or achieve euvolemia despite adequate and escalating doses of diuretics (ter Maaten et al., 2015). While the expected diuretic and natriuretic responses to 40 mg of furosemide are thought to be around 3–4 L per day and 200–300 mmol/L, respectively, up to 40% of hospitalized patients with HF fail to do so (Valente et al., 2014), and in the context of a cardiorenal syndrome, these doses will hardly achieve these diuretic outputs.

Several mechanisms have been classically described as causes of an impaired diuretic response. Among these, we have: 1) a reduced delivery of diuretic to the kidney’s proximal tubule due to reduced cardiac output, which constitutes the cause of the increasingly high diuretic doses required in HF; 2) compensatory sodium reabsorption either after the diuretic effect wears off or at other nephron segments, something that has been described in healthy adults but seems not to occur in patients with AHF (Cox et al., 2021); and 3) congestive nephropathy, where the increased sympathetic tone causes a chronic redistribution of blood into the central circulation, leading to a rise in intraabdominal pressure, which in turn increases renal venous pressure, decreases renal blood flow, glomerular filtration, and, therefore, urine output (Husain Syed et al., 2021).

In any case, once congestion begins, volume overload will progressively worsen HF signs and symptoms by adding more workload to the heart and increasing renal vein pressures, thus initiating a vicious cycle where diuretic resistance leads to prolonged lengths of stay and increased mortality (O’Connor et al., 2011). Therefore, it is crucial to identify these patients rapidly, as they could benefit from more aggressive and individualized treatment.

## 5. Diagnostic Tools in Congestion

Congestion must be evaluated clinically in patients at risk. Although clinical history and physical exam are the first steps in congestion assessment, other tools can help us identify subclinical congestion, quantify its severity, and provide treatment monitoring and follow-up measures (Adamson et al., 2014).

### 5.1. Anamnesis and Physical Exam

On anamnesis, we need to identify dyspnea, orthopnea, bendopnea, and paroxysmal nocturnal dyspnea. Also, inquiring about the abdominal or ankle perimeters is critical (McDonagh et al., 2021). Less frequent symptoms are nocturnal cough, loss of appetite, bloated feeling, confusion, depression, dizziness, and syncope.

Physical signs of congestion are based on detecting increased filling pressures or extravascular fluid overload. The more specific signs are elevated jugular venous pressure, hepatojugular reflux, third heart sound, and laterally displaced apical impulse (McGee, 1998; McDonagh et al., 2021). One of the most useful physical findings is the jugular venous pulse, which detects systemic congestion and elevated left-sided filling pressures (McDonagh et al., 2021).

However, clinical signs of congestion do not have a high sensitivity and are usually evident in advanced states. In some series of patients with chronic HF, physical signs of congestion were absent in up to 42% of patients with the Pulmonary Capillary Wedge Pressure (PCWP) > 22 mmHg (Stevenson and Perloff, 1989).

### 5.2. Biomarkers

A clinically helpful biomarker should possess the following characteristics: it should be quickly determined, affordable, and provide additional information not attainable from the clinical interrogatory or physical examination alone. There is no current definitive biomarker that fulfills all these requisites; however, in this section, we will discuss the ones available or under development.

Clinical guidelines suggest measuring natriuretic peptides in all patients with AHF. These have a high negative predictive value for HF and congestion as causes of dyspnea (Ponikowski et al., 2016), with thresholds for ruling out AHF of Brain Natriuretic Peptide (BNP) < 100 pg/ml and N-terminal prohormone of BNP (NT-proBNP) < 300 ng/ml, which may vary according to sex, ejection fraction, and the presence of atrial fibrillation. They are elevated due to increased ventricular filling pressures, often defined as hemodynamic or *intravascular congestion*, while not necessarily tissular congestion (e.g., edema, crackles) (de la Espriella et al., 2022a). The possible reliance of natriuretic peptides on renal clearance has motivated some discussion about their clinical utility in patients with a severely reduced estimated glomerular filtration rate (eGFR) (de la Espriella et al., 2022b). One of the differences between both natriuretic peptides relies on their elimination and half-life. BNP is degraded by enzymatic processes and has a short lifespan in circulation of approximately 20 min. On the other hand, NT-proBNP is eliminated renally and has a longer half-life of around 120 min, hence its higher blood values. With this reasoning, BNP used to be the recommended natriuretic peptide to be measured in the setting of renal dysfunction. However, it has been demonstrated that both are equally unreliable markers in patients with AKI or unstable worsening renal function (WRF) (Koratala and Kazory, 2017). They could probably have a role in showing improvement trends at low eGFRs as long as they remain stable. However, the recommended cut-off in patients with an eGFR <60 ml/min/1.73 m^2^ to keep a sensibility close to 90% and a specificity of 72% is a remarkably high value of 1,200 ng/ml (Anwaruddin et al., 2006). To date, there is no evidence that natriuretic peptides have a significant correlation with congestion in patients on dialysis treatment; therefore, their use is not recommended in this population (Koratala and Kazory, 2017). Given all these caveats, there is an important limit to the information and usefulness of NT-proBNP in the setting of renal dysfunction, and, although normal values can help discard congestion or HF, high values are dependent on the patients’ eGFRs and, therefore, they are suboptimal biomarkers in cardiorenal syndrome.

Antigen carbohydrate 125 (CA125), a glycoprotein synthesized by celomic epithelium in pleura, pericardium, or peritoneum, is well-known as a biomarker for some malignancies, such as ovarian cancer. Recently, it has been identified as a reliable biomarker for congestion (Núñez et al., 2014). Among the favorable characteristics associated with this rediscovered biomarker are its low price, long half-life (up to a week), and that it is unaffected by renal dysfunction (Núñez et al., 2020a; de la Espriella et al., 2022a). The pathophysiology of CA125 elevation in congestion is not well-established. Nevertheless, it has been hypothesized that it is due to the activation of mesothelial cells due to hydrostatic pressure increase, mechanical stress, and inflammatory cytokines (Huang et al., 2012). Recent evidence demonstrated a good positive and improved correlation with NT-proBNP, with other *tissular congestion* markers, such as pleural effusion, ascites, elevated jugular venous pressure, hepatomegaly, and leg edema (Núñez et al., 2020a; de la Espriella et al., 2022a). The cut-off of 35 U/ml has been used to guide treatment and has been associated with improved eGFR compared to those treated by clinical guidance alone at 72 h post-admission (Núñez et al., 2020b). Currently, two clinical trials evaluated a therapeutic strategy guided by CA125 concentration, contrasting it with classic management guided by signs and symptoms with promising results (Núñez et al., 2016; Núñez et al., 2020b).

It is essential to highlight the role of plasma creatinine in clinical practice, as an increase of this parameter could reflect parenchymal damage in the kidney or hemodynamic changes (e.g., hyperfiltration correction). Thus, if decongestion is being achieved and there is clinical improvement, modest rises in serum creatinine are expected and should not result in treatment suspension or dose reduction, as this phenomenon is not associated with renal damage (McCallum et al., 2021). On the other hand, a lack of clinical improvement or adequate diuresis is associated with ominous outcomes, and nephrology should be consulted (Emmens et al., 2022).

Hemoconcentration has been seen after diuretic treatment, but studies revealed a weak association, making it a poor marker for decongestive therapy (Darawsha et al., 2016). Soluble CD146 (Gayat et al., 2015) and adrenomedullin (Kremer et al., 2018) are other novel biomarkers that can offer additional information for cardiac congestion, but their use is currently restricted to research (Ambrosy et al., 2013).

### 5.3. Imaging

#### 5.3.1 Chest X-Ray

Chest X-ray has been an essential tool in the diagnosis of congestion. It can show signs of vascular redistribution towards the upper lobes, upper pulmonary veins’ distention, hilar structures’ enlargement, or septal lines in the lower lung (Kerley A and B lines). Moreover, pleural fluid accumulation in right HF leads to the thickening of interlobar fissures or pleural effusion. Another radiologic finding frequently detected in congestion is cardiomegaly. If congestion increases up to alveolar edema, chest X-rays show bilateral opacities with central distribution and no air bronchogram. These radiologic signs can precede clinical symptoms’ onset and may be visible for days after successful decongestion (Cardinale et al., 2014).

#### 5.3.2 Multi-Organ Ultrasound

Although the findings mentioned above in chest X-rays are common, up to 20% of patients with clinical congestion have a normal chest X-ray (Collins et al., 2006). Lung ultrasound has recently emerged as a more reliable tool in ruling out interstitial edema or pleural effusion. The echogenicity of the lung is related to the amount of water in the interstitial space. Thus, lung ultrasound detects B-lines originating from fluid in the interstitium and alveoli (Al Deeb et al., 2014). At least three B-lines in two fields bilaterally have a sensitivity of 94%–97% and specificity of 96%–97% to detect congestion in AHF (Pivetta et al., 2019). Lung-ultrasound-guided diuretic treatment of pulmonary congestion has proven to reduce the number of decompensations and improve the functional capacity of patients with HF (Rivas ‐ Lasarte et al., 2019). Moreover, diagnosis of pleural effusion with lung ultrasound is also an easily acquired skill that may not only be useful for diagnosis but also as guidance for diagnostic or therapeutic thoracentesis. Thus, as a non-invasive, safe, and easy-to-use technique, lung ultrasound may have an important role in congestion diagnosis and management.

Echocardiography, used as a bedside tool, can estimate right- and left-sided filling pressures. Assessment of inferior vena cava (IVC) and its collapsibility and width estimates right atrial pressure (RAP) and left ventricular filling pressures (Berthelot et al., 2020). Thus, an IVC diameter lower than 1.5 cm and collapsibility >50% is a good estimate for RAP <5 mmHg. On the other hand, a diameter >2.5 cm and no collapsibility estimate a RAP >20 mmHg (Pellicori et al., 2021).

**FIGURE 2:**
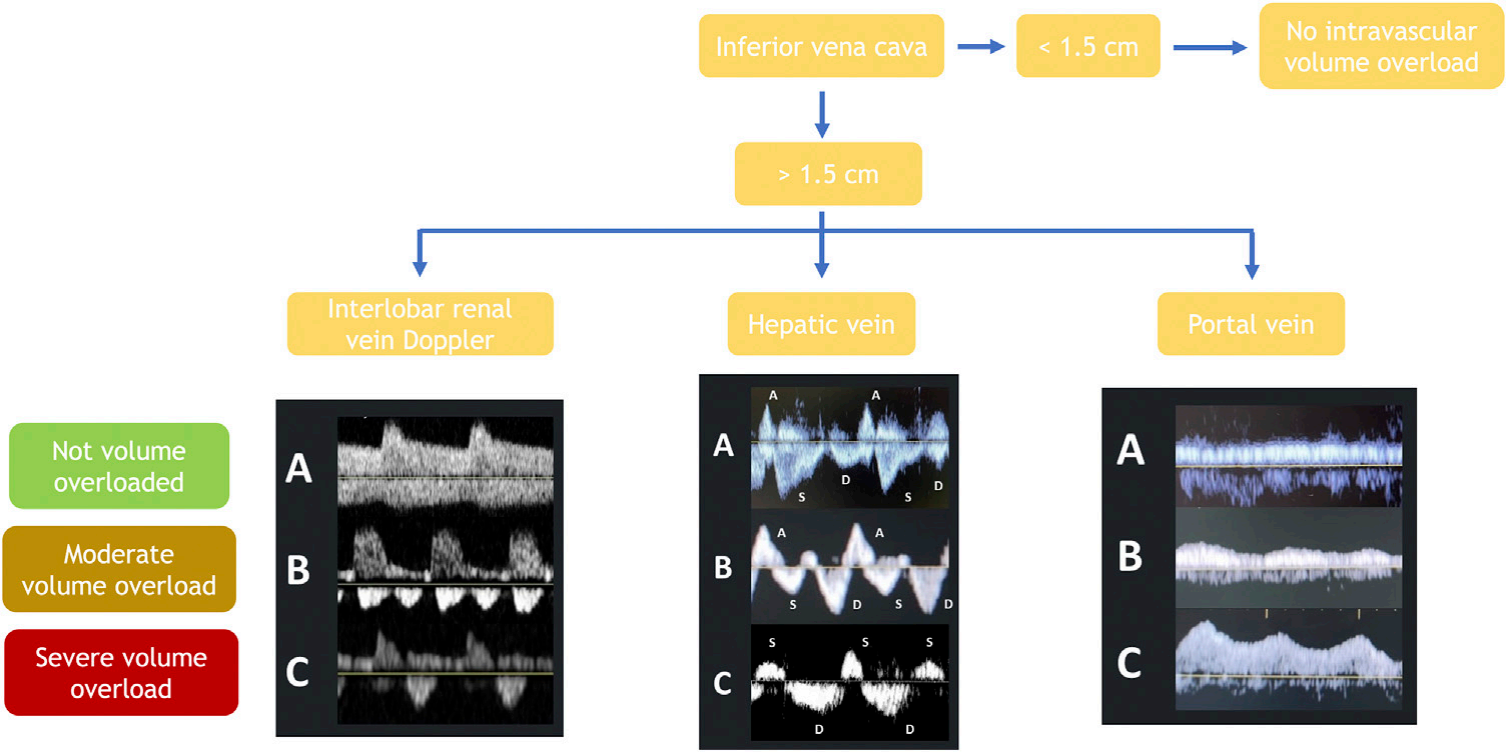
Abdominal ultrasound-guided diagnosis of volume status and its severity by assessing the inferior vena cava’s diameter, followed by Doppler evaluation of portal, hepatic and renal interlobar vein waves.

When the IVC is enlarged, one should assess the hepatic veins. These veins are thin-walled and communicate with the IVC. Although the right and middle veins are the most readily observed, any of the three hepatic veins (right, middle, or left) can be measured. When applying the pulsed Doppler on the hepatic vein of healthy subjects, the tracing observed should show a small retrograde A wave and two antegrade waves: first, the larger wave called S wave corresponding to systole and then a smaller one called D wave corresponding to diastole (panel A, Figure 2) (Beaubien-Souligny et al., 2020). In volume overload, RAP will increase, causing the S wave’s magnitude to progressively decrease, first becoming smaller than the D wave (panel B, Figure 2) and later becoming retrograde (panel C, Figure 2). However, volume overload and other causes of increased RAP will generate this phenomenon (e.g., tricuspid regurgitation) (Argaiz et al., 2021).

Unlike the hepatic veins, the walls of the portal vein are thick. When applying pulsed Doppler over the portal vein of healthy subjects, a continuous flow should be observed (panel A, Figure 2). In a state of volume overload, the pressure in the vein increases, causing the flow to become pulsatile (panel B, Figure 2) and then biphasic in cases of severe congestion (panel C, Figure 2) (Argaiz et al., 2021; de la Espriella et al., 2022a). The portal vein study is of great value in cases where the IVC or hepatic veins cannot be evaluated due to confounding factors (e.g., tricuspid insufficiency and mechanical ventilation). A case in which its study will not be valuable is in patients with liver cirrhosis or other severe hepatic interstitial pathology.

Cardiac Doppler imaging and tissue Doppler can assess left-sided filling pressures (Mullens et al., 2009). When filling pressures increase, diastolic mitral inflow velocities change with an increase in early velocities (high E-wave with short deceleration time and low A-wave; E/A ratio >2). Oppositely, tissue Doppler velocities decrease with a low e’ (59). Thus, ratio E/e’ > 15 indicates a restriction in diastole and elevated left-sided filling pressures. These assumptions have some limitations in daily practice. Diastolic mitral inflow velocities cannot be correctly assessed in patients with atrial fibrillation, a frequent pathology in patients with AHF. Moreover, e’-wave has limitations in patients with prosthetic mitral valves or significant mitral annulus diseases such as degenerative calcification.

Recommendations for the evaluation of left ventricular diastolic function by echocardiography were updated in 2016 by the American Society of Echocardiography (ASE) and the European Association of Cardiovascular Imaging (EACVI) (Nagueh et al., 2016). Despite the large number of parameters that can be evaluated with echocardiography, both in HF with preserved (HFpEF) and reduced ejection fraction (HFrEF) a simplified scheme is proposed including: E/e’ ratio >14, left atrium volume >34 ml/m^2^, tricuspid regurgitation velocity >2.8 m/s and e’ septal velocity <7 cm/s or e’ lateral velocity <10 cm/s. Patients with at least two previous conditions are likely to have diastolic dysfunction with chronic exposition to high left-side filling pressures.

Ultrasound evaluation of renal blood flow in HF has become another valuable tool for congestion diagnosis and management (Nijst et al., 2017). The objective of renal venous ultrasound is to observe the interlobar or arcuate arteries and veins located in the renal cortex. As they pass together, they can be distinguished as blue and red pulsatile flows with the color Doppler. In euvolemic subjects, a continuous wave should be observed below the arterial pulsatile wave (panel A, Figure 2). In cases of volume overload, this continuous wave will become biphasic (panel B, Figure 2), and in cases of severe congestion, it becomes monophasic (panel C, Figure 2) (Nijst et al., 2017). As the renal blood flow is part of the systemic circulation, it may be altered by other causes of increased RAP and volume overload (e.g., tricuspid regurgitation or high intrabdominal or intraparenchymal renal pressure in ascites or obstructive uropathy) (Argaiz et al., 2021).

Two indexes have been developed to quantify venous renal flow modifications secondary to elevated central venous pressures. The first, the venous impedance index (VII), quantifies the velocity changes in renal venous flow during the cardiac cycle (if congestion increases, the index approaches 1). The second one, the venous discontinuity index (VDI), quantifies the time without blood flow in interlobar veins (high when a single flow phase in diastole is observed) (Pellicori et al., 2021).

The role of this technique in congestion management has hardly been studied. One study evaluated the effect of volume loading and diuretics on renal venous flow pattern observing that intravascular expansion resulted in significant blunting of venous flow before a substantial increase in cardiac filling pressures could be demonstrated (*via* IVC diameter). Moreover, impaired renal venous flow was correlated with less diuretic efficiency, and patients with a lower VII had a better diuretic response (Nijst et al., 2017). Thus, this parameter might be an early marker of congestion development and become helpful in treating congestion prematurely.

There is a lack of evidence in the renal-ultrasound-guided diuretic treatment of congestion; a future field for further investigations and its usefulness in individuals with advanced CKD is unknown.

Internal jugular vein (IJV) ultrasound has been related to the classical jugular vein distention (JVD) sign and is a volume or pressure overload marker. Clinical evaluation might be subjective and challenging in some patients, but ultrasonography allows for identifying and quantifying this phenomenon. It should be performed with the head and neck elevated 45° and carefully to avoid IJV compression. If it is difficult to visualize, asking the patient to cough or perform a Valsalva will allow to identify it. The JVD ratio is the difference between IVJ at rest and during Valsalva. When congestion worsens, IJV diameter at rest increases. Thus, a JVD ratio <4 is abnormal, and if congestion is severe, it can decrease to <2 (Pellicori et al., 2021). A low JVD ratio is related to severe symptoms and elevated natriuretic peptides (Pellicori et al., 2014) and predicts increased HF hospitalizations or deaths (Pellicori et al., 2015).

#### 5.3.3 Others

Bioelectrical impedance analysis (BIA) measures the impedance of the body to an alternating electric current of known characteristics, this being the result of two variables: Resistance (R) and Reactance (Xc). The BIA measures the resistance of the whole body resembling a homogeneous cylinder. Though there are several commercially available devices with different characteristics (bioimpedance spectroscopy (BIS), single-frequency BIA, multifrequency-BIA (MF-BIA), bioelectrical impedance vector analysis (BIVA)), to determine the amount of intra- and extracellular water indirectly and thus estimate the degree of overhydration of congestive patients. Its results are not reliable if the patient has metal objects such as prostheses or major amputations (although in the case of BIS, results can be adjusted), and it is contraindicated if the patient has a pacemaker or self-implantable defibrillator in the case of MF-BIA, BIVA, BIS or segmental BIA (Moissl et al., 2013). Moreover, it must be considered that some devices can detect the third-space volume while others do not.

### 5.4. Invasive Measurements

Right heart catheterization (RHC) has long been considered the gold standard to diagnose the presence of increased intracavitary filling pressures, including RAP and PCWP and for the measurement of pulmonary vascular resistances (Bootsma et al., 2022). These measures play a pivotal role in diagnosing HFpEF (Pieske et al., 2019). RHC is also mandatory for diagnosing pulmonary hypertension (Simonneau et al., 2019) and for the workup of patients considered for heart transplantation or implantation of a left ventricular assist device (Mehra et al., 2016; Guglin et al., 2020).

RHC use in AHF decreased considerably after the publication of the ESCAPE trial, which did not show a benefit of RHC-guided therapy for patients admitted for decompensated heart failure compared with usual care; this trial, however, did not include patients in cardiogenic shock (Hill et al., 2005). Interestingly, RHC appears to be gaining ground, particularly in the setting of cardiogenic shock, where hemodynamic profiling of patients has been associated with lower in-hospital mortality in observational studies (Garan et al., 2020), and as part of a team-based approach, where decisions to institute mechanical circulatory support based on RHC data may improve outcomes in this patient population (Tehrani et al., 2019). There are some caveats in the use of right heart catheterization. It is invasive and not available in everyday clinics and only provides information on the specific moment when it is performed.

Nevertheless, wireless pulmonary artery hemodynamic monitoring has shown promising results in HF patients with previous HF hospitalizations to detect subclinical congestion, leading to anticipated decongestive therapies that significantly decrease HF hospitalizations, regardless of left ventricular ejection fraction (Givertz et al., 2017; Shavelle et al., 2020). The use of this device is on the rise, having recently received expanded FDA approval for HF patients with NYHA II functional class and elevated natriuretic peptides (NP). The indication was supported by the results of the GUIDE HF trial, where after adjustment for COVID-19 impact on the trial results, patients in NYHA class II and elevated NP had a reduction in the composite primary endpoint of reduced mortality and HF events (defined as HF hospitalizations and urgent visits when their therapy was guided by hemodynamic monitoring (Lindenfeld et al., 2021).

## 6. Management

**FIGURE 3:**
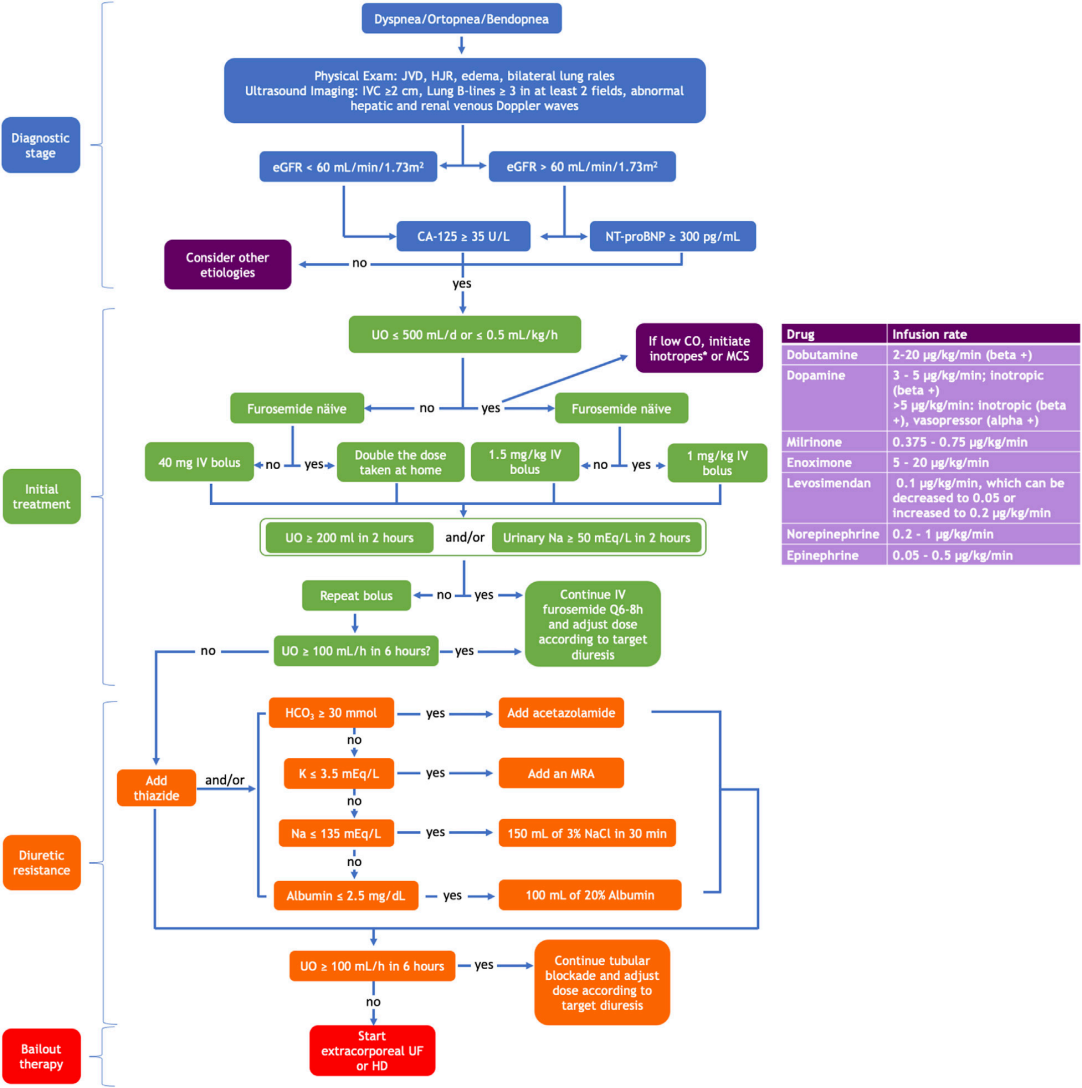
A multimodal approach using clinical assessment, biomarkers, and imaging tools to diagnose heart failure and kidney disease congestion and its stepwise therapeutic algorithm. CO, cardiac output; eGFR, estimated glomerular filtration rate; HCO_3_, bicarbonate; HD, hemodialysis; HJR, hepatojugular reflux; IV, intravenous; IVC, inferior vena cava; JVD, jugular vein distention; K, potassium; MCS, mechanical circulatory support; MRA, mineral receptor antagonist; Na, sodium; NaCl, sodium chloride; UF, ultrafiltration; UO, urinary output; *Adapted from the ESC Guidelines for the diagnosis and treatment of chronic heart failure 2021.

The key to managing cardiorenal syndrome should always be to solve the root issue: heart failure or kidney disease. However, most times, the root problem does not have a solution, or if it has one, it is not instantaneous. In such cases, physicians must manage congestion, which derives from either organ’s dysfunction. Herein, we discuss pharmacological and extracorporeal therapies that have alleviated congestion, reduced readmissions, and sometimes mortality. A multimodality diagnostic and treatment algorithm is proposed in Figure 3.

### 6.1. Diuretics

Most patients who present with an acute cardiorenal syndrome are volume overloaded. The only way to remove the excess fluid pharmacologically is by blocking sodium and water reabsorption at the kidney tubules with diuretics (Table 1). When the kidneys cannot increase urine output and achieve decongestion, we find ourselves with a case of diuretic resistance (Wilcox et al., 2020).

**TABLE 1:** Most frequently used diuretics. Type, site of action, dose, absorption, onset, and duration of action.

Diuretic type	Frequently used	Site of action	Oral absorption	Route of administration	Dose	Onset	Peak of action	Duration
Loop diuretics	Furosemide	Ascending loop of Henle	10—100%	Oral	20 mg QD—200 mg TID	30—60 min	1—2 h	6—8 h
				IV	20 mg QD—2g QD	5—10 min	0.5 h	2 h
	Bumetanide		80—100%	Oral	0.5 mg QD—5 mg TID	0.5—1 h	1—2 h	4—6 h
				IV		2—3 min	15—30 min	2—3 h
	Torsemide		80–100%	Oral	10 mg QD—100 mg TID	1 h	1—2 h	6—8 h
Thiazide and thiazide-like diuretics	Hydrochlorothiazide	Early distal tubule	65—75%	Oral	25 mg QD—50 mg QID	2 h	4 h	6—12 h
	Chlorthalidone		-	Oral	25 mg QOD—50 mg BID	2.6 h	2—6 h	24—72 h
	Metolazone		65%	Oral	2.5–20 mg QD	1 h	-	24 h
Mineralocorticoid receptor antagonists (MRA)	Spironolactone	Late distal tubule	90%	Oral	12.5 mg QD—50 mg BID	-	2.6—4.3 h	48—72 h
	Eplerenone		69%	Oral	25—50 mg QD	-	1.5—2 h	-
Epithelial sodium channel (ENaC) blockers	Amiloride	Late distal tubule	30—90%	Oral	5 mg QD—BID	2 h	6—10 h	24 h
Carbonic anhydrase inhibitors	Acetazolamide	Proximal tubule	Dose dependent	Oral	250 mg QD—500 mg TID	1—2 h	8—18 h	8—24 h
				IV	500 mg QD—TID	2—10 min	15 min	4—5 h
Sodium-glucose co-transporter 2 inhibitors (SGLT2i)	Dapagliflozin	Proximal tubule	78%	Oral	10 mg QD	-	2 h	72 h
	Empagliflozin		-	Oral	10 mg QD	-	1.5 h	72 h
Vasopressin antagonists	Tolvaptan	Collecting duct	56%	Oral	15—60 mg QD	2—4 h	4—8 h	Dose dependent

IV, intravenous; QD, once a day; BID, twice a day; TID, three times a day; QOD, every other day; QID, four times a day; h, hours; min: minutes

#### 6.1.1 Loop Diuretics

In a healthy individual, kidneys filter 180 L of plasma a day. Most water reabsorption occurs thanks to the sodium-potassium-chloride cotransporters (NKCC) in the loop of Henle, which reabsorb sodium in high quantities, increasing the solute concentration at the kidney’s medulla (Novak and Ellison, 2022). This osmotically potent medulla will allow most of the filtered water to be reabsorbed by the aquaporins in the collecting duct. This countercurrent effect disappears when the loop’s channels are blocked, increasing urine output significantly. These drugs’ most notable side effects are hypokalemia, hypomagnesemia, hypochloremia (with the consequent metabolic alkalosis), and volume depletion.

##### 6.1.1.1 Which One Should be Used?

Furosemide is the most used and cheap loop diuretic; unfortunately, its oral bioavailability is highly variable, ranging from 10 to 100%. More modern loop diuretics like bumetanide and torsemide have a higher and more consistent oral bioavailability between 80 and 100%. However, torsemide is of particular interest as it has a longer half-life than furosemide and bumetanide (3.5 vs. 1.5 h), and its effect can last up to 16 h. Moreover, torsemide has proven to significantly reduce HF symptoms by improving the NYHA functional class, increasing cardiovascular survival, and non-significantly reducing heart failure-related hospital admissions (Abraham et al., 2020). Moreover, there is evidence that torsemide can produce aldosterone inhibition (Tsutamoto et al., 2004), potentially impacting cardiac remodeling (Yamato et al., 2003). There are two ongoing clinical trials, TORNADO (The Impact of Torsemide on hemodynamic and Neurohormonal Stress, and cardiac remodeling in Heart Failure) (NCT01942109) and TRANSFORM-HF (Torsemide comparison with furosemide for management of Heart Failure) (NCT03296813), that put this hypothesis to the test.

Furthermore, furosemide’s bioavailability can be affected by many factors, such as other medications, delayed gastric emptying, reduced systemic perfusion, or gut edema. Acute congestion can produce the last two. Hence, the most efficient way to deliver enough loop diuretics in circulation in acute congestion is intravenously. There is no clear data on which is the proper initial furosemide dose in cases of AHF (Mullens et al., 2019). Recently, Rao et al. (2021) have proposed the implementation of a natriuretic response prediction equation (NRPE) to guide loop diuretic treatment in HF patients by assessing urinary sodium output, calculated according to the equation described in the article, 2 h after receiving a loop diuretic and increasing the dose if urinary sodium output is suboptimal (<50 mmol).

Similarly, a post-hoc analysis of the ROSE-AHF trial associated urinary sodium at the first void <60 mmol with longer hospital stays and lower weight loss. Nevertheless, the clinical application of this “suboptimal urinary sodium excretion” needs to be better defined, as some measure it in the first 6 h, others in the first hour, and others in the first void. Likewise, the urinary sodium threshold is not well defined either, though it is usually 50—60 mmol (Tersalvi et al., 2021). However, it is known that high diuretic doses improve dyspnea, reduce weight, and increase net fluid loss without worsening long-term renal outcomes (Felker et al., 2011; Brisco et al., 2016).

The suggested initial furosemide dose in the ESC position paper on diuretic use in HF (Mullens et al., 2019) recommends starting with 40 mg of furosemide or an equivalent if the patient is diuretic naïve, or if not, then 1 to 2 times the dose they were taking at home. However, we suggest that there should be a distinction if the patient is oliguric or anuric. If this is the case, we recommend initiating the furosemide stress test, which has been validated in the oliguric AKI setting (Chawla et al., 2013). It consists of administering a 1 mg/kg (∼60 mg) bolus if the patient is diuretic naïve or a 1.5 mg/kg (∼100 mg) bolus if the patient is on chronic diuretic therapy. With either method, if the diuretic response in 2 h is equal to or greater than 150–200 ml, the next dose should be adjusted and administered in the following 6–8 h, according to the initial diuretic response observed. Contrarily, if the urine output in the next 2 h is less than 150–200 ml, then a new bolus of the same dose should be immediately administered. If diuresis continues to be less than 150–200 ml in 2 h, then the patient is considered non-responsive to intravenous furosemide and could benefit from a sequential diuretic blockade approach (Gill et al., 2021).

In patients who do respond to intravenous loop diuretics, there are conflicting data on whether it is better to administer the drug in a continuous infusion or bolus (Cox et al., 2020). The DOSE trial (Felker et al., 2011) found no difference in symptoms between patients who received bolus vs. continuous infusion; however, patients in the continuous group did not receive an initial bolus, which could have delayed the time until the drug reached threshold levels, potentially affecting the results.

##### 6.1.1.2 Subcutaneous Diuretic Infusion

Furosemide has been reformulated for subcutaneous administration to allow an “intravenous-like” diuretics delivery in out-hospital patients.

Nevertheless scarce, there is evidence that subcutaneously administered furosemide for AHF treatment (Gilotra et al., 2018; Birch et al., 2021). This route allows patients to (Pitt et al., 1999; Zannad et al., 2011) be treated at home and can reduce HF hospitalizations, safely as no WRF, ototoxicity, or skin irritation has been reported as a consequence of this treatment (Ojeifo et al., 2016; Sica et al., 2018). Despite the potential advantages seen with this therapy, it has not been incorporated into management guidelines. More evidence should come in this area in the following years.

#### 6.1.2 Thiazide and Thiazide-Like Diuretics

Thiazide diuretics act on the sodium-chloride cotransporter (NCC) located in the distal convoluted tubule. The most used thiazide diuretics are hydrochlorothiazide, metolazone, and chlortalidone. There is also available an intravenous thiazide, chlorothiazide. These agents are primarily utilized in the clinical setting as an antihypertensive medication rather than diuretics *per se*. However, in combination with a loop diuretic, they can significantly increase natriuresis and improve congestion by acting as a sequential diuretic (Cox et al., 2022). It should be noted that chlorthalidone and metolazone are slowly absorbed, and their first dose should be given around 8 h before administering a loop diuretic (Mullens et al., 2019).

Though these drugs were thought not to be effective in patients with an eGFR below 30 ml/min/1.73 m^2^, there is compelling evidence of their effectiveness in advanced CKD. For instance, chlortalidone has been proven to increase diuresis and improve blood pressure control in advanced CKD while significantly reducing albuminuria, rendering a nephroprotective effect (Agarwal et al., 2021). Currently, there is an ongoing study (NCT03574857) comparing metolazone and chlorothiazide in diuretic resistance in the context of AHF. These drugs’ most important side effects are hypokalemia, hyponatremia, hypochloremia, and hyperuricemia (Novak and Ellison, 2022).

#### 6.1.3 Mineralocorticoid Receptor Antagonists

Mineralocorticoid receptor antagonists (MRA) act by inhibiting the action of aldosterone in the principal cells of the connecting and collecting tubule, reducing the number of epithelial sodium channels (ENaC). They have a weak diuretic effect, though their efficacy is significantly augmented in cases of hyperaldosteronism related to HF or cirrhosis. Three main agents belong to this class, spironolactone, eplerenone, and, in the immediate future, finerenone.

They have a more delayed onset of action than the rest of the mentioned diuretics, requiring 2 or 3 days until any effect can be seen. Blocking sodium reabsorption creates a lumen-positive electrical gradient that impedes potassium and hydrogen secretion, thus potentially leading to hyperkalemia and acidosis. Therefore, they should be used cautiously in patients with CKD stage 3b or greater. However, they are quite useful in the setting of HF as part of the sequential tubular blockade, given that this side effect balances the risk of hypokalemia and metabolic alkalosis produced by the concomitant use of loop and thiazide diuretics.

Moreover, this drug class is of particular interest given that they serve as diuretics and are also cardioprotective. The RALES (Pitt et al., 1999) and EMPHASIS-HF (Zannad et al., 2011) studies have proven that both eplerenone and spironolactone reduce cardiovascular events in patients with HFrEF. In the TOPCAT trial (Pitt et al., 2014), spironolactone did not benefit patients with HFpEF, though there are doubts about adherence to the medication in the eastern European individuals included in this study (de Denus et al., 2017). Furthermore, finerenone has reduced the number of cardiovascular and kidney events in patients with diabetic kidney disease (Bakris et al., 2020; Pitt et al., 2021; Agarwal et al., 2022), though their diuretic potency has not been assessed yet.

#### 6.1.4 Epithelial Sodium Channel (ENaC) Blockers

Amiloride and Triamterene act similarly to MRA though instead of indirectly blocking ENaC channel expression by blocking aldosterone, they directly inhibit ENaC function. Amiloride is currently recommended over triamterene as it is better tolerated, and it can be administered once a day instead of twice daily. It does not have triamterene’s side effect of crystalluria, leading to cast formation and triamterene stones (Sica and Gehr, 1989). Given that these agents do not block aldosterone, they do not have the added cardioprotective benefit, so MRAs are recommended above this class.

#### 6.1.5 Acetazolamide

Acetazolamide, a carbonic anhydrase inhibitor, works in the proximal convoluted tubule by blocking the sodium–hydrogen exchanger 3 (NHE3), inhibiting bicarbonate and sodium reabsorption, causing metabolic acidosis. Its diuretic effect may be minimal due to the multiple distal compensation mechanisms; however, like in the case of MRA, this side effect is desirable to balance the metabolic alkalosis produced by the urinary chloride losses caused by both loop and thiazide diuretics (Verbrugge et al., 2019). In fact, it has an intense diuretic effect when combined with other diuretic classes, although tachyphylaxis has been described after 72 h of use, as alkalosis is corrected.

In addition, acetazolamide has been associated with renal vasodilation due to the increased sodium that reaches the *macula densa*, similar to the effect seen with SGLT2 inhibitors, which could be nephroprotective. Moreover, it can also improve apnea-hypopnea symptoms associated with central sleep apnea, a common disorder found in HF patients (Gill et al., 2021).

An ongoing multicenter clinical trial (NCT03505788) will evaluate if the addition of intravenous acetazolamide 500 mg once daily to loop diuretics adds clinical benefit in acutely decompensated HF patients.

#### 6.1.6 Sodium Glucose Cotransporter 2 Inhibitors (SGLT2i)

SGLT2i also act at the proximal convoluted tubule by blocking the sodium-glucose cotransporter 2. This way, they reduce glucose reabsorption by 30%–50% (Narasimhan et al., 2021) and increase natriuresis reducing volume overload and improving tension control by reducing preload (Lytvyn et al., 2017). The current members of this family are empagliflozin, canagliflozin, dapagliflozin, ertugliflozin, and sotagliflozin. There is compelling evidence that these agents reduce cardiovascular events in both diabetic (Zinman et al., 2015; Neal et al., 2017) and non-diabetic patients with HF with reduced (McMurray et al., 2019; Packer et al., 2020) and preserved ejection fraction (Anker et al., 2021) in the chronic and acute settings.

In addition to these beneficial cardiac effects, they have also proven to slow CKD progression in both diabetic (Perkovic et al., 2019) and non-diabetic patients (Heerspink et al., 2020). The natriuretic, cardiovascular, and renal beneficial effects of this drug family make them particularly attractive for patients with CRS. Moreover, the recently published EMPULSE study determined the safety and usefulness of empagliflozin in managing acute decompensated HF (Voors et al., 2022). The only agents that have proven beneficial in renal and cardiovascular outcomes in the absence of type 2 diabetes are dapagliflozin and empagliflozin (McMurray et al., 2019; Heerspink et al., 2020; Packer et al., 2020; Butler et al., 2022). For its part, empagliflozin seems beneficial independent of the left ventricular ejection fraction (Packer et al., 2020; Butler et al., 2022). Therefore, they should be introduced during the HF admission and maintained at discharge if tolerated well. As with RAAS inhibitors, a drop in eGFR <30% is expected and should not lead to its withdrawal.

#### 6.1.7 Vasopressin Receptor 2 Antagonists

Vaptans act by inhibiting vasopressin receptor type 2 at the collecting duct, the nephron’s last site of water reabsorption (Greenberg and Verbalis, 2006), making them an attractive target in volume overloaded HF patients. These agents are widely used to treat hyponatremia, autosomal dominant polycystic kidney disease (Torres et al., 2018), and inappropriate antidiuretic hormone secretion syndrome (Burst et al., 2017). Despite achieving a faster improvement in weight loss and edema, no mortality or readmission benefit was seen with tolvaptan in AHF in the EVEREST trial (Konstam et al., 2007). This negative result was confirmed by two additional trials (Felker et al., 2017; Konstam et al., 2017). The absence of long-term beneficial effects could mean that natriuresis is more important in AHF than mere free-water excretion (Narasimhan et al., 2021). Nevertheless, these agents could help manage refractory congestion in the presence of hyponatremia in selected patients.

#### 6.1.8 Diuretic Resistance

##### 6.1.8.1 Why Does Diuretic Resistance Occur?–Do Not be Afraid of High Doses

Like all other diuretics except for MRA, loop diuretics circulate in the blood bound to albumin and are, therefore, not filtered by the kidney. They can then act on the NKCC channel placed in the loop’s lumen only after being secreted by a transporter in the proximal tubule in competition with other molecules such as urea. This means that in cases of acute tubular necrosis or reduced nephron mass, there are fewer transporters available to secrete these diuretics into the lumen, which is why in cases of AKI or CKD, higher diuretic doses are required to allow for enough drug to make its way to the channel we want to block.

The reduced systemic perfusion and renal blood flow in AHF mislead the kidneys into wanting to reabsorb as much volume as possible, increasing the number of transporters in the proximal and distal tubule. When the loop NKCC transporter is blocked, the concentration of sodium and chloride that reaches the distal tubule in the presence of an increased number of transporters enhances the amount of water reabsorbed at this level (Narasimhan et al., 2021). This is the basis for the sequential or segmental diuretic therapy approach (Narasimhan et al., 2021; Cox et al., 2022).

For many years, compensatory post-diuretic sodium reabsorption (CPSR) was thought to be the main reason for intravenous loop diuretic resistance. CPSR was described in healthy individuals as a decrease in renal sodium secretion after the loop diuretic level drops to concentrations lower than its threshold (Kelly et al., 1983). This phenomenon was not only recently disproven to participate in the development of diuretic resistance in AHF, but that those patients who have a greater diuretic and natriuretic response to furosemide present a larger post-diuretic spontaneous diuresis (Cox et al., 2021).

### 6.2. Sequential Diuretic Tubular Blockade

The renal tubule consists of four main segments, the proximal convoluted tubule, the loop of Henle, the distal tubule, and the collecting duct. The main concept of this approach is that when the loop diuretic effect is insufficient to achieve decongestion, we must block the rest of the tubular transporters.

#### 6.2.1 Proposed Sequential Diuretic Tubular Blockade

There is evidence that a multi-diuretic drug sequential blockade regimen benefits roughly 60% of patients with diuretic resistance (Cox et al., 2022). Our proposed approach in the acute setting is explained in Figure 3. In the outpatient setting, on top of the indicated therapy according to the patients’ ejection fraction, we suggest they could benefit from an oral loop diuretic (preferably torsemide). If insufficient, oral metolazone or chlortalidone may be considered (it could be administered every other day, given its long half-life). Finally, if these diuretics are insufficient or the patient becomes alkalotic (bicarbonate >30 mmol/L) or hypokalemic (potassium <3.5 mmol/L), oral acetazolamide and MRAs could be added. We do not recommend adding amiloride or tolvaptan due to the lack of long-term cardiovascular benefits.

### 6.3. Additional Treatment to Diuretic Therapy

#### 6.3.1 Inotropic Agents

Inotropic agents can be of use in cases of hypoperfusion due to low cardiac output syndromes that lead to low renal blood flow, sodium retention, and less diuretic delivery to the proximal tubule. In general terms, current guidelines restrict the use of inotropes for the treatment of HF patients who are hypotensive or hypoperfused since they have been otherwise associated with a worse long-term prognosis (Nagao et al., 2022). Inotropes aim to increase cardiac output by enhancing cardiac contractility, and they are considered the third pharmacological pillar in decompensated HF treatment after diuretics and vasodilators (Farmakis et al., 2019). Currently, three classes of inotropes are recommended for decompensated HF: beta-adrenergic agonist (dobutamine, epinephrine, and norepinephrine), phosphodiesterase III inhibitor (milrinone), and calcium sensitizers (levosimendan) (Farmakis et al., 2019). Selecting the proper agent in each situation can be challenging.

Dobutamine, a beta-adrenergic inotrope, has a renal sympathetic activity that increases renal blood flow and the glomerular filtration rate but impairs oxygenation of the medulla, increasing the oxygen demand in the kidney tissue (Al-Hesayen and Parker, 2008). Milrinone, a phosphodiesterase III inhibitor, induces vasodilation, enhancing trans-renal perfusion pressure and increasing renal blood flow and renal oxygen delivery without significant glomerular filtration rate changes. In the end, for any beneficial renal effect to occur, the mean arterial pressure needs to be maintained to ensure proper renal perfusion pressure. This can be achieved with the administration of vasopressors such as norepinephrine (Zima et al., 2020), though trial results have only found limited beneficial results with these agents (Cuffe et al., 2002).

Levosimendan has been used to facilitate the weaning of continuous inotropes, augment diuresis in cardiorenal syndrome, and as cardiogenic shock therapy in selected patients (Yeung et al., 2021). Various lines of clinical investigation have produced indications of a net beneficial impact of levosimendan on renal dysfunction (Mebazaa et al., 2007). Apart from improving left ventricular performance, levosimendan effects include pre-glomerular vasodilation, increased artery diameter, and renal blood flow (Yilmaz et al., 2013). Compared to dobutamine in the LIDO trial (Follath et al., 2002), levosimendan was associated with an increase in the glomerular filtration rate.

In the past, dopamine was thought to increase renal blood flow and urinary sodium excretion; nevertheless, the addition of low-dose dopamine (2 mcg/kg/min) to diuretic treatment in patients with AHF and renal dysfunction has not shown significant effects on urine volume or renal function, and it is no longer used for this purpose (Ungar et al., 2004).

Therefore, in the setting of a low cardiac output-induced cardiorenal syndrome, we propose that levosimendan may be the first inotrope treatment option (Farmakis et al., 2019).

#### 6.3.2 Intravenous Albumin

Albumin has been broadly prescribed for critically ill patients, although it has no known mortality benefit. It increases intravascular oncotic pressure and produces fluid mobilization from the interstitium to the intravascular space, which is thought to improve diuresis. The hypothesis that co-administration of furosemide and albumin can achieve a better diuresis response than diuretics alone has been debated. In theory, given that furosemide travels albumin-bound in the circulation, good renal perfusion and albumin are required for furosemide to arrive and be secreted at the tubular lumen of the proximal tubule. Hence hypoalbuminemia could decrease furosemide diuretic efficacy. Different trials showed inconsistencies in published results on this topic. A retrospective analysis (Doungngern et al., 2012) in intensive care unit patients with continuous furosemide infusion therapy did not show significant differences in mean urine output in patients with albumin co-administration. On the other hand, a randomized controlled crossover study (Phakdeekitcharoen and Boonyawat, 2012) in stable hypoalbuminemic CKD patients demonstrated superior short-term efficacy of albumin co-administration over furosemide alone in enhancing water diuresis and natriuresis. It is important to highlight the population and methods differences of these previous studies that might explain the results differences. A recent meta-analysis revealed that albumin co-administration increased urine output by 31.45 mL/h and urine sodium excretion by 1.76 mEq/h compared to furosemide alone (Lee et al., 2021). This effect was better in patients with low baseline serum albumin levels (<2.5 g/L) and high albumin infusion dose (>30 g) and within 12 h after administration. Diuretic and natriuretic effects were better in patients with mildly impaired renal function. Nevertheless, further clinical trials are needed to examine outcomes due to limited enrolled participants. In view of this data, we suggest the co-administration of albumin and furosemide only in cases of diuretic resistance and moderate-severe hypoalbuminemia (2–2.5 mg/dL).

#### 6.3.3 Hypertonic Saline Infusion

The combination of hypertonic saline infusion, ranging from 1.4% if serum sodium greater than 136 and 4.6% if lesser than 125, with high-dosed furosemide has been proposed to mitigate renal dysfunction and promote natriuresis (Paterna et al., 2011). A meta-analysis demonstrated that in patients with advanced HF, concomitant hypertonic saline administration improved weight loss, preserved renal function, and decreased length of hospitalization, mortality, and HF rehospitalization (Gandhi et al., 2014). Similarly, real-world analysis in patients with refractory AHF (Griffin et al., 2020) showed that the administration of 150 mL of 3% sodium chloride in 30 min improved urine output, weight loss, serum sodium, chloride, and creatinine concentrations. Diuretic efficiency, defined as the change in urine output after doubling the diuretic dose, also improved. The mechanism involved is not fully understood, though it is believed that not only sodium but chloride plays a crucial role in salt-sensitive renal responses and acts on the family of WNK kinases which regulate the transporters where loop and thiazide diuretics act (Griffin et al., 2020). An ongoing clinical trial will measure the effects of chloride supplementation in cases of AHF (Mechanism and Effects of Manipulating Chloride Homeostasis in Stable Heart Failure; NCT03440970).

Despite the wide heterogeneity between different analyses, and the lack of an adequately powered, multi-center, randomized, blinded trial, we believe that hyponatremic and hypochloremic patients with diuretic resistance may benefit from the co-administration of hypertonic saline infusion and intravenous diuretics.

#### 6.3.4 Neprilysin Inhibitors

Natriuretic peptides such as atrial natriuretic peptide (ANP) or brain natriuretic peptide (BNP) are cardiac hormones. ANP exerts diuretic, natriuretic, and vasodilatory effects that help maintain water-salt balance and regulate blood pressures by reducing preglomerular vascular resistance stimulating diuresis and natriuresis (Tersalvi et al., 2020). Pleiotropic effects on cardiac homeostasis have also been described as pro-angiogenetic, anti-inflammatory, and anti-atherosclerotic (Forte et al., 2019). However, their biological function is impaired in HF patients due to neprilysin-mediated degradation. Treatment with sacubitril/valsartan, first-line therapy in HFrEF, reduces the degradation of natriuretic peptides by inhibiting neprilysin and inhibits the renin-angiotensin-aldosterone system. This combination reduces cardiovascular mortality (McMurray et al., 2014) and adverse myocardial remodeling and slows down WRF (Damman et al., 2018). Additionally, treatment with sacubitril/valsartan has been associated with a higher reduction of congestive clinical signs and less diuretic intensification (Selvaraj et al., 2019). Hence sacubitril/valsartan could be considered an interesting therapeutic tool in the outpatient setting to maintain euvolemia and avoid congestion in patients with HFrEF without deleterious effects on renal function (Witteles et al., 2007; Owan et al., 2008; Dandamudi and Chen, 2012).

### 6.4. Ultrafiltration

#### 6.4.1 Peritoneal Dialysis

Peritoneal dialysis (PD) is mainly known as a renal replacement therapy technique. However, it has also been used as a tool for volume removal for more than 50 years. It consists of the infusion of osmotically active solutions in the peritoneal cavity, where these solutions ultrafiltrate both free-water and sodium through the peritoneal membrane (Teitelbaum, 2021). The most frequently utilized solutions rely on glucose to induce the osmotic gradient necessary for ultrafiltration. However, glucose-based solutions require longer dwell times in the peritoneal cavity to efficiently remove plasma sodium, increasing glucose absorption by the patient and injuring the peritoneal membrane with glucose end-products (Zemel et al., 1994). In this sense, icodextrin (a glucose polymer) allows for higher sodium removal and longer dwell times without the harmful side effects of glucose-based solutions, thus improving sodium balance and patients’ metabolic profile. There is evidence that, in patients with HF and CKD, PD improves the quality of life, reduces hospital readmissions, helps preserve renal function (Courivaud et al., 2014), maintains patient autonomy as it can be performed at home, and is also beneficial in patients with right-sided HF, pulmonary hypertension, and ascites (Lu et al., 2015). In addition, some reports show that left ventricular ejection fraction can slightly improve after initiating PD (Morales et al., 2021). Recovery of ventricular and renal function may be explained by the better management of congestion and the prescription of the standard of care pharmacological treatments that are often withdrawn from patients with CKD due to the risk of hyperkalemia. Moreover, PD has also been associated with removing inflammatory cytokines such as interleukin-1, -6, and TNF-α, which could induce cardiac and renal fibrosis (Zemel et al., 1994).

In summary, despite the lack of clinical trials evaluating PD effect on mortality and other hard cardiovascular outcomes, we recommend it as a valuable option in autonomous patients with CKD and frequent readmissions due to AHF. This therapy allows them more independence, renal function preservation, and improved quality of life with fewer HF hospital readmissions (Lu et al., 2015).

#### 6.4.2 Extracorporeal Ultrafiltration

Given the side effects and limitations of diuretic treatments, there has been growing interest in a non-pharmacological management approach to congestion. There is conflicting evidence on whether ultrafiltration (UF) brings any benefit on top of a proper diuretic regimen. The CARESS-HF trial by Bart et al. (2012) tried to answer this question by recruiting patients with AHF and then assigning them to diuretic therapy targeting a urine output of 3–5 L per day or venovenous fixed UF at a rate of 200 mL/h with the Aquadex System 100. There was no difference in weight loss between groups, though there was a rise in serum creatinine, bleeding events, risk of initiating renal replacement therapy, and catheter-related complications in the UF group (Bart et al., 2012).

However, there are reports showing that UF slightly reduces rehospitalizations within 30 days of an acute decompensated HF episode and helps achieve greater weight reduction if an individualized rather than a fixed UF rate is used. For instance, in the CUORE trial (Marenzi et al., 2014), the UF rate was adjusted according to each participant’s clinical needs without exceeding 75% removal of the weight gained. This study found that despite weight reduction being similar in both groups, patients who received UF had fewer hospital admissions up to 6 months after being discharged from the hospital. The AVOID-HF (Costanzo et al., 2016) and UNLOAD trials (Costanzo et al., 2007) have also reported fewer hospital HF readmissions after using UF devices. To date, there is no data on whether UF has any mortality benefit over intravenous diuretic therapy; however, the ongoing PURE-HF trial (NCT03161158) is reassessing this dilemma by evaluating cardiovascular mortality and HF events at 90 days after discharge in patients managed with tailored UF in addition to low-dose diuretics vs. intravenous diuretics alone.

In summary, isolated UF is useful, particularly in patients who have trouble achieving sufficient decongestion with diuretic therapy, though one should not forget the implications of such a therapy. There could be bloodstream catheter-associated infections and potential bleeding complications derived from the anticoagulation required to perform this technique.

## 7 Discussion and Future Directions

The interplay between the heart and the kidney has been a matter of concern for a long time. Still, it has gained interest recently, leading to the constitution of cardiorenal units with a multidisciplinary approach. Recent advances have highlighted the need for the pre-clinical and multiparameter diagnosis of congestion, with the integrated use of biomarkers and bedside ultrasound. Multiple treatment strategies have been studied: diuretics in different doses or combinations have been the cornerstone of congestion treatment, but newer drugs and less conventional pharmacological and non-pharmacological approaches are becoming available to clinical practice. The lack of strong scientific evidence for many of these strategies contrasts with the clinical need to implement them for the increasingly diagnosed refractory congestion.

There is an urgent need for collaborative research in the field of heart and kidney failure, especially in the setting of congestion. Better diagnostic techniques to identify a pre-clinical state may lead to anticipated and effective treatment. A multidisciplinary approach, led by cardiology and nephrology, will eventually answer the needs of this increasing patient population.
